# Experimental Investigation of the Drying Shrinkage Performance of a Modified Ceramsite Geopolymer Concrete

**DOI:** 10.3390/ma18040915

**Published:** 2025-02-19

**Authors:** Peng Deng, Xuening Wang, Jian Guo, Yan Liu, Qi Zheng

**Affiliations:** 1 College of Civil Engineering and Architecture, Shandong University of Science and Technology, Qingdao 266590, China; dengpeng1226@sdust.edu.cn (P.D.); 202282040038@sdust.edu.cn (X.W.); guojian119@aliyun.com (J.G.); 202082040056@sdust.edu.cn (Q.Z.); 2 Shandong Provincial Key Laboratory of Civil Engineering Disaster Prevention and Mitigation, Shandong University of Science and Technology, Qingdao 266590, China

**Keywords:** modified ceramsite geopolymer concrete (MCGC), pretreatment, silicone resin modification, drying shrinkage, compressive strength, predictive model

## Abstract

The experiments were divided into two groups to establish a drying shrinkage model suitable for modified ceramsite geopolymer concrete (MCGC). In the first experimental group, via comparison with dry ceramsite (untreated), a method for modifying the ceramsite surface with a 6% silicone resin was proposed which could reduce its water absorption, enhance the compressive strength and slump of the corresponding concrete, and decrease the drying shrinkage. The second group systematically explored the influences of control factors on MCGC prepared from modified ceramsite. Different water/binder (w/b) ratios, [Na_2_O]/b ratios, and metakaolin content (MK/b) ratios were used in the experiment. Compressive strength and drying shrinkage tests were performed for 90 d. High w/b and Na_2_O/b ratios could enhance drying shrinkage. Moreover, 8% Na_2_O/b enhanced the compressive strength. Low compressive strength was obtained using 10% Na_2_O/b. A high MK/b ratio reduced drying shrinkage. However, high w/b and MK/b ratios hindered strength development. Finally, a model predicting drying shrinkage for MCGC with a high prediction accuracy was proposed by considering three control factors. Due to the variety of ceramsite pretreatment methods and the considered factor limitations, the model had potential for additional enhancements.

## 1. Introduction

Ceramsite concrete is lightweight aggregate concrete with relatively high strength, good heat preservation, and good thermal insulation; thus, it merits extensive application in engineering [1,2]. Portland cement is a common raw material for preparing conventional ceramsite concrete. Nevertheless, its production consumes an enormous amount of energy and generates large amounts of carbon dioxide (CO_2_) [3]. As a new type of cementitious material, geopolymer, produced by using slag, metakaolin (MK), fly ash, and other materials at room temperature or a slightly higher temperature, offers the potential to solve these problems due to its low energy consumption levels, low CO_2_ emissions, and superior performance characteristics [4,5,6]. The application of geopolymers has been widely studied in the production of concrete. Therefore, it is highly valuable to use geopolymers instead of Portland cement to prepare ceramsite concrete. However, studies have shown that an increase in binder content increases the level of drying shrinkage [7,8,9]. Moreover, due to the special attributes of aggregates, the volume stability of ceramsite concrete is relatively poor, and its drying shrinkage is usually greater than that of conventional concrete [10,11,12,13]. The drying shrinkage of concrete may lead to a decrease in strength and durability, which further affects the safety and use of the structure. Nevertheless, most of the existing studies on drying shrinkage are aimed at geopolymer concrete or ceramsite concrete. Whether such concrete is suitable for a lightweight aggregate geopolymer concrete is unknown. Hence, it is necessary to explore the development characteristics and factors influencing lightweight aggregate geopolymer concrete shrinkage and provide references for its practical application.

Currently, to reduce the drying shrinkage of lightweight aggregate, pretreatment is usually performed. Among them, prewetting treatment is a very common method in engineering [14,15]. When the concrete is dried and dehydrated, the adsorbed water stored in the lightweight aggregate can supplement it, delaying the increase in capillary pressure, thereby reducing the drying shrinkage [16]. However, although this method is simple to use, it is inefficient, and the durability of concrete cannot be guaranteed. To solve this problem, the use of an appropriate modifier [17,18,19] for the surface mentioned in the study of recycled aggregate is an alternative method. Katz [20] introduced the technique of soaking recycled aggregate with silica fume solution, and the results showed that the strength of concrete was improved. This treatment is to coat silica fume on the surface of recycled aggregate, which enhances the structure of the aggregate and improves the strength of concrete. Ismail and Ramli [21] used a calcium metasilicate solution to modify the surface of recycled aggregate and reduced the drying shrinkage of the corresponding concrete by 26% at 28 d compared with that of the untreated aggregate. In general, this method improved the quality of the aggregate and slowed down the drying shrinkage stress. Noticeably, recycled aggregate has greater water absorption, which is similar to that of the lightweight aggregate. This result arises because the recycled aggregate in concrete produces additional interfacial transition zones with high porosities [22].

Furthermore, previous studies have indicated that geopolymer concrete generally has a greater drying shrinkage than conventional concrete [23]. The shrinkage behavior of geopolymers is affected by multiple factors, such as the water/binder (w/b) ratio, activator solution modulus, alkali concentration, and cementitious material content [24,25]. Among these factors, the w/b ratio plays a key role. Sadeghian et al. [26] reported that when the alkali-activated slag concrete’s w/b ratio is large, additional pores are generated due to water evaporation, thereby increasing shrinkage deformation. Ou et al. [27] and Yang et al. [28] similarly concluded that increasing w/b ratio leads to increased drying shrinkage. Moreover, the modulus of the activator solution (SiO_2_/Na_2_O molar ratio) and the alkali concentration are important factors affecting shrinkage. Hu et al. [29] reported that as the SiO_2_/Na_2_O molar ratio elevates from 0 to 1.5, the autogenous shrinkage of alkali-activated slag mortars first elevates and then decreases, and the drying shrinkage rate gradually increases. Neto et al. [30] reported the effect of the sodium oxide content (Na_2_O/b ratio) on slag geopolymer concrete’s shrinkage behavior, indicating that at a SiO_2_/Na_2_O molar ratio of 1.7, the concrete’s total shrinkage increases with elevating Na_2_O/b ratio, and the drying shrinkage increases significantly. The reason is due to the reduction in total porosity and extremely fast pore size refinement. Moreover, adding cementitious materials can moderate geopolymer concrete’s drying shrinkage. Yang et al. [31] used 0~30% fly ash instead of metakaolin to study the effects of fly ash content on metakaolin-based geopolymer concrete’s shrinkage and found decreased drying shrinkage and increased autogenous shrinkage. These results arise because adding fly ash increases the refinement of the cementitious system, increases the capillary pressure, and limits water evaporation. Samson et al. [32] obtained similar results. Applying alkali-activated composites greatly restricts concrete’s drying shrinkage [33]. In addition, the development of a time-dependent drying shrinkage prediction model is also of critical importance for alkali-activated concrete. However, research in this area remains notably scarce. Studies have indicated that the CEB-10 model significantly underestimates the shrinkage of alkali-activated slag concrete, and the JSCE-15 model is similarly inadequate in predicting its shrinkage behavior [34]. Mastali et al. [35] investigated the predictive discrepancies among the ACI-209, B3, and GL2000 models, revealing substantial differences between the predictions of B3 and GL2000, while the ACI-209 model demonstrated better alignment with experimental data. Due to the complexity of concrete material properties and mix proportion parameters, as well as the variability in the reaction processes involved in drying shrinkage, the establishment of a predictive model necessitates the consideration of a broader range of parameters.

In summary, the integration of modified lightweight aggregate with geopolymer concrete not only maximizes the inherent thermal insulation and excellent seismic performance of lightweight aggregate concrete but also highlights the eco-friendly properties of geopolymer concrete. Thus, this research proposes combining different pretreatment methods (prewetting and silicone resin modified surfaces) of ceramsite specimens with mixtures of different control factors (w/b, Na_2_O/b, and MK/b ratios) to prepare modified ceramsite geopolymer concrete (MCGC) and investigate their effects on the workability, compressive strength, and drying shrinkage behaviors. On the basis of the test results, a high-accuracy model predicting drying shrinkage of MCGC is developed.

## 2. Experimental Procedures

### 2.1. Mixture Design

To systematically explore ceramsite geopolymer concrete’s drying shrinkage, two experimental groups were designed. Table 1 presents the concrete’s mix. First, in group 1, different ceramsite treatment methods were used before mixing, and the effect of the silicone resin was relatively good. Then, based on this method, the second group explored the development characteristics of drying shrinkage by comparing different parameter values. The specific experimental design was as follows.

In group 1, the ceramsite was prewetted and modified with silicone resin and compared with dry ceramsite (untreated). The ceramsite’s water absorption and drying shrinkage rates were measured, and the slump and compressive strength tests were performed to evaluate its engineering performance. In this group, the mix proportion was based on the existing research results of our academic team. In group 2, seven series of concrete specimens were assembled with three w/b ratios (0.40, 0.45, and 0.50), Na_2_O/b ratios (6%, 8%, and 10%), and MK/b ratios (30%, 50%, and 70%), each to explore the development characteristics. To simplify the experiment, other factors were controlled in each series to take conservative values to study the influence of a single variable. Through a large number of trials in the early stage of the test, the 33% sand ratio effectively maintained the fluidity of the mixture and could make the concrete strength reach the expectation. Therefore, for all the concrete mixtures, the sand ratio was 33%, and the constant SiO_2_/Na_2_O molar ratio was 1.5.

### 2.2. Materials

**Figure 1:**
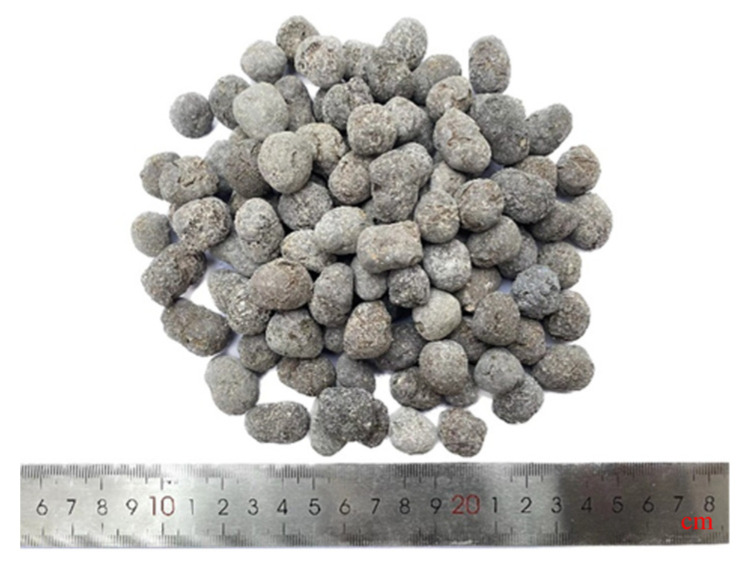
Spherical shale ceramsite.

**Figure 2:**
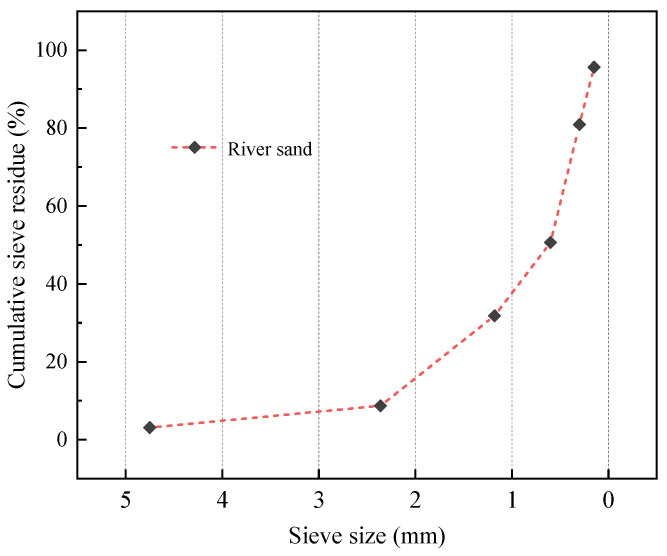
Grading curve for the fine aggregate.

As shown in Figure 1, the coarse aggregate utilized here was spherical shale ceramsite produced by Shandong Lanling Hongxian Building Materials Technology Co., Ltd. (Linyi, China). Table 2 summarizes the measurement of shale ceramsite’s physical properties. The modifier was a silicone resin, and its technical indexes are provided by manufacturers in Beijing, China, as shown in Table 3. The fine aggregate utilized was river sand with a fineness modulus of 2.6, and its bulk and appearance densities were 1550 and 2720 kg/m^3^, respectively. Figure 2 shows the grading curve of the fine aggregate. Slag and MK were used as binder materials. The slag’s specific surface area was 472 m^2^/kg and the density was 2.84 g/cm^3^. The MK’s specific surface area was 713 m^2^/kg and the density was 3.98 g/cm^3^. Table 4 shows the main chemical compositions of the two specimens according to the manufacturers.

Water, liquid sodium silicate (water glass), and sodium hydroxide with >99% purity were utilized to prepare the activators. The modulus of the water glass was 3.34, the density was 1.387 g/cm^3^, and the glass contained 65.00 wt.% H_2_O, 27.60 wt.% SiO_2_, and 7.30 wt.% Na_2_O. In addition, to avoid the decrease in concrete strength caused by the hydration reaction between traditional water reducers (such as polycarboxylate superplasticizers) and alkali activators, calcium sucralose (water-reducing retarder) was used in this experiment.

### 2.3. Testing

In this test, a total of 48 specimens (100 mm × 100 mm × 100 mm) were designed and tested using a compression machine (Wuxi Jianyi Instrument Machinery Co., Ltd., Wuxi, China) according to the Chinese standard GB/T 50081-2019 [36]. The compressive strengths at 3, 7, and 28 d were measured by loading the specimens at a constant speed of 0.5 MPa/s. To ensure the accuracy of the results, three specimens of each mix proportion were measured, and the mean value was calculated. Notably, since this study involved nonstandard specimens, the results were multiplied by 0.95 (i.e., the size conversion coefficient).

A concrete shrinkage dilatometer was utilized to measure 30 specimens (100 mm × 100 mm × 515 mm), and the mean drying shrinkage rate of all mix proportions was calculated on the basis of the Chinese standard GB/T 50082-2009 [37]. Each specimen was subjected to three individual tests. A sensor head was embedded into two sides of each specimen. The initial lengths of the specimens were measured when the curing age was 3 d, and the specimens were subsequently incubated at 20 ± 2 °C in a closed space with 60 ± 5% relative humidity. Following moving the specimens into the closed space, the lengths of the specimens were measured at 1, 3, 7, 14, 28, 45, 60, and 90 d. Before testing, the dial gauge was zeroed using a standard rod.

## 3. Results and Discussion

### 3.1. Effects of Different Ceramsite Treatment Methods on Concrete (Group 1)

#### 3.1.1. Water Absorption

**Figure 3:**
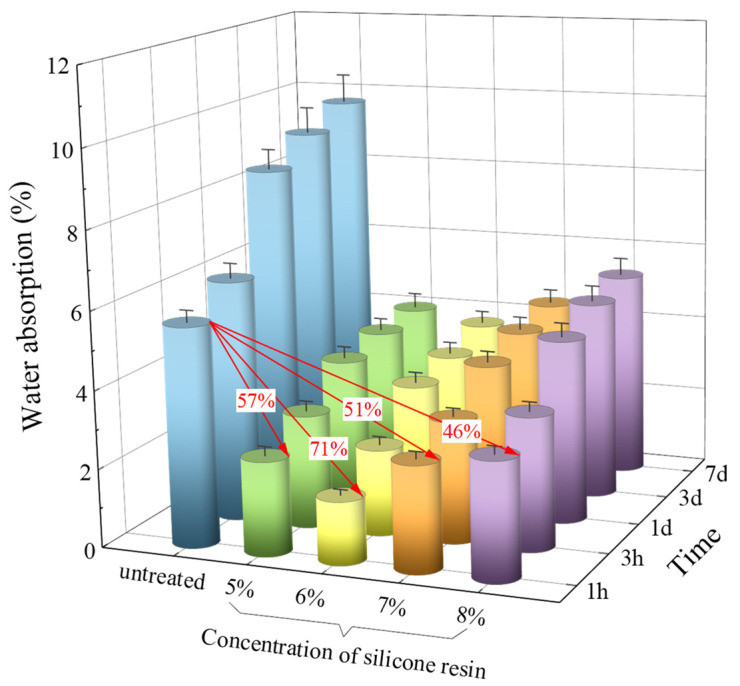
Water absorption of ceramsite treated by different methods.

As displayed in Figure 3, the 24 h water absorption of ceramsite modified with 5%, 6%, 7%, and 8% silicone resin was 2.4%, 1.6%, 2.7%, and 3.0%, respectively, which was about 57%, 71%, 51%, and 46% lower than that of untreated ceramsite. This showed that the ceramsite’s water absorption was significantly reduced after modification, and a low water absorption was achieved in the specimen with 6% silicone resin. This was potentially due to the fact that silicone resin could effectively seal the surface pores of the ceramsite and improve its hydrophobicity [38]. However, the modified film formed by high concentrations of silicone resin encapsulated some water, thereby increasing the weight of the ceramsite and the level of water absorption [39].

#### 3.1.2. Slump

**Figure 4:**
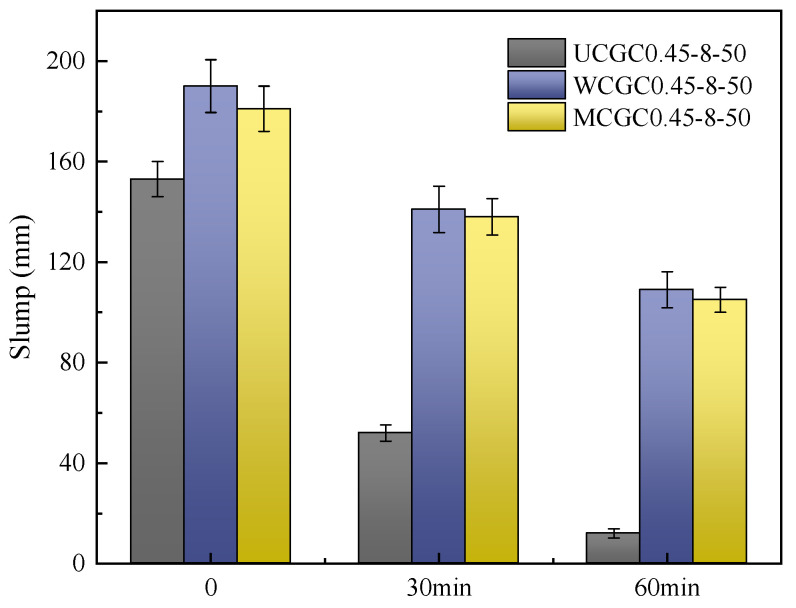
Slump levels of the concretes with different ceramsite pretreatments.

Figure 4 shows the results of the slump test for concrete with different pretreated ceramsite contents. Clearly, pretreatment with ceramsite could significantly increase the workability and reduce the slump loss. The slump loss levels of UCGC0.45-8-50 at 30 and 60 min reached 101 mm and 141 mm, respectively. Compared with those of UCGC0.45-8-50, the slump loss levels of WCGC0.45-8-50 and MCGC0.45-8-50 at 60 min decreased by approximately 43% and 42%, respectively. This was potentially due to a layer of organic molecular film on the surface of the treated ceramsite, which effectively reduced the absorption of water, thereby improving the fluidity of the concrete [40].

#### 3.1.3. Compressive Strength

**Figure 5:**
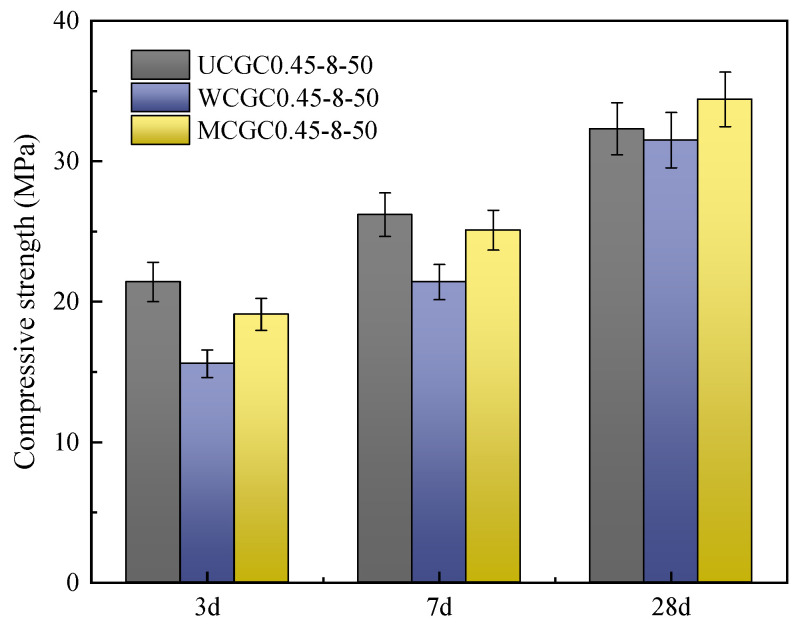
Compressive strengths of the concretes with different ceramsite pretreatments.

As shown in Figure 5, the compressive strength of WCGC0.45-8-50 at 3 d, 7 d, and 28 d was 15.6 MPa, 21.4 MPa, and 31.5 MPa, respectively, which was about 27%, 18%, and 2% lower than that of UCGC0.45-8-50, respectively, indicating that the prewetting treatment weakened the concrete’s compressive strength. This was potentially due to the prewetted ceramsite releasing water during hydration and forming a water film between the aggregate and concrete slurry, reducing the interfacial bonding strength [41]. Conversely, the compressive strength of MCGC0.45-8-50 decreased slightly or even increased after the ceramsite was modified by silicone resin. This finding was similar to those of previous studies [42]. Silicone resin had a certain hydrophobicity, preventing the water film formation and increasing the concrete’s strength.

#### 3.1.4. Drying Shrinkage

**Figure 6:**
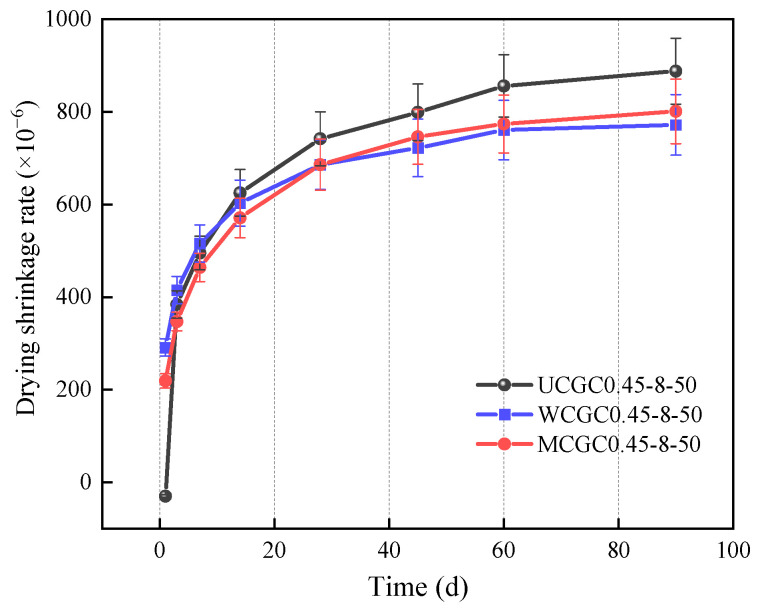
Drying shrinkage of concrete with different pretreated ceramsite materials.

The concrete’s drying shrinkage is crucial for evaluating the long-term performance of concrete’s structure [43]. Figure 6 displays the effects of different pretreatment methods of ceramsite on geopolymer concrete’s drying shrinkage characteristics. The results indicate that the drying shrinkage rates were significantly elevated before 7 d and slowly increased between 7 and 90 d for all the mixtures. UCGC0.45-8-50, which contained dry ceramsite, exhibited relatively great drying shrinkage after 7 d. The main cause was that the dry ceramsite absorbed large amounts of water during preparation, leading to large pores in the concrete [44,45]. In addition, the drying shrinkage of WCGC0.45-8-50 peaked before 7 d, but the growth rate decreased significantly after 7 d. This was potentially due to the prewetted ceramsite increasing the total w/b ratio, enhancing the evaporation of moisture [16]. The moisture released by the ceramsite during hardening could counteract drying shrinkage while accelerating the hydration of cementitious materials to reduce capillary stress, thus improving the specimen’s drying shrinkage [46].

The 28 d and 90 d drying shrinkage of MCGC0.45-8-50 was 8% and 10% less than that of the untreated specimen, respectively. Therefore, the modification of ceramsite by silicone resin significantly controlled the concrete’s drying shrinkage. This phenomenon could arise due to the high hydrophobicity of the modified film, which significantly reduced water evaporation-induced drying shrinkage. Furthermore, the presence of silicone resin molecules ameliorated the pore structure, serving as a reason for mitigating drying shrinkage [42]. Notably, compared with PCGC0.45-8-50, MCGC0.45-8-50 exhibited a decrease in drying shrinkage of approximately 16% and 10% after 3 and 7 d, respectively. The findings demonstrated that the silicone resin modified surface enhanced the volume stability of the concrete.

### 3.2. Effects of Different Factors on the MCGC (Group 2)

Based on the test results of group 1, ceramsite modified with silicone resin improved the workability of the corresponding concrete to a certain extent and was conducive to increasing the strength and improving the drying shrinkage behavior. Therefore, follow-up tests were performed using 6% silicone resin.

#### 3.2.1. Compressive Strength

**Figure 7:**
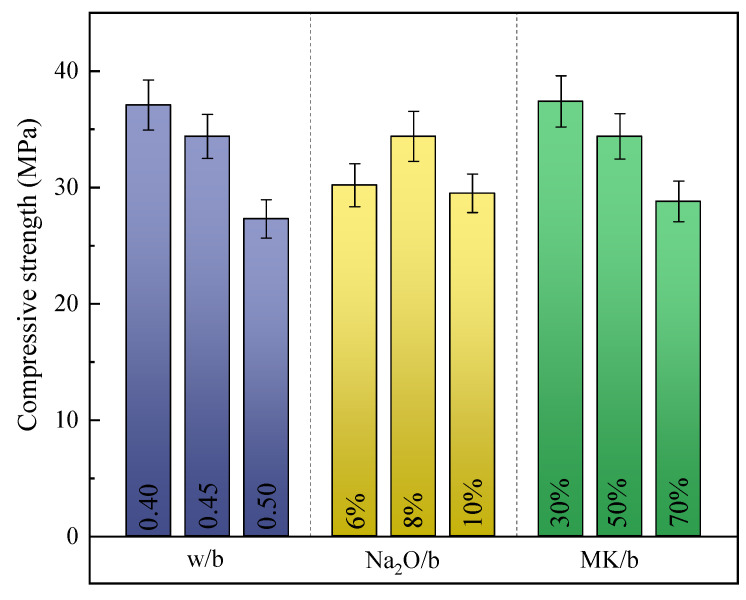
Compressive strength of MCGC at 28 d with different w/b ratios, Na_2_O/b ratios, and MK/b ratios.

During the observation of the compressive strength testing procedure, it was discernible that the compressive failure modes of the MCGC exhibited a consistent pattern. As the load was applied, cracks emerged at the superior and inferior edges of the specimens and propagated towards the central region. Subsequently, the slurry that covered the surface of the specimens detached, accompanied by the fragmentation of the internal ceramsite particles. Figure 7 displays MCGC’s compressive strength at 28 d, which indicated its reduction with the elevated w/b ratio. Increasing the w/b ratio from 0.4 to 0.5 resulted in around 26% reduced compressive strength, being consistent with prior findings [47,48]. The elevated w/b ratio increased water evaporation from pores, thereby increasing the porosity and weakening concrete’s compressive strength [49,50,51]. Additionally, an increase in the MK/b ratio adversely affected MCGC’s compressive strength. When the MK/b ratio was increased from 30% to 50%, the compressive strength was reduced by about 23%. The current results were consistent with these studies [52,53]. This result was simply attributed to the increased N-A-S-H colloids with large mesh pores [54]. Figure 7 displays that as the Na_2_O/b ratio elevated to 10%, MCGC’s compressive strength first elevated and then reduced. Similar findings have also been documented previously [55]. When the Na_2_O/b ratio was 8%, MCGC’s compressive strength was the largest, which was 34.4 MPa, due to the acceleration of the destruction–condensation period. However, a Na_2_O/b ratio that was overly high could lead to early precipitation of the gel to inhibit the continued release of the monomer [56]. Furthermore, according to the literature [57], excessive Na decreased the microstructural density, which was part of the reason for the decreased compressive strength.

#### 3.2.2. Drying Shrinkage

**Figure 8:**
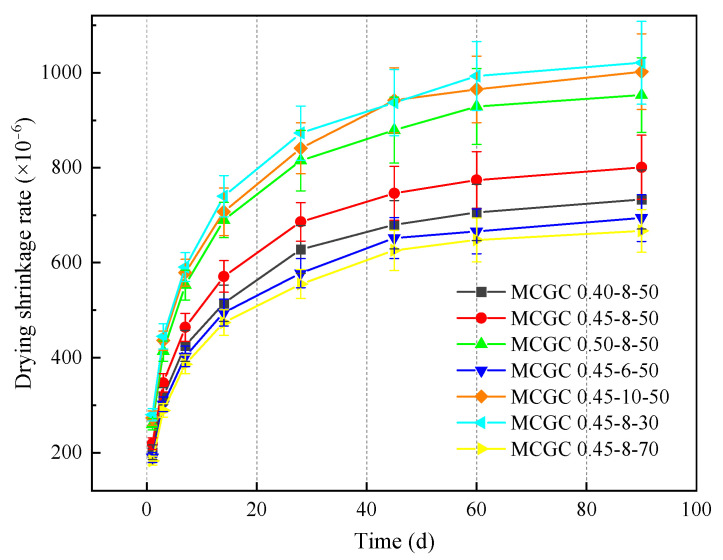
Drying shrinkage development of MCGCs with various w/b, Na_2_O/b, and MK/b ratios.

Figure 8 shows the drying shrinkage development over time for MCGC with various w/b, Na_2_O/b, and MK/b ratios. For all the MCGC groups, the drying shrinkage increased over time. However, shrinkage mainly occurred before 60 d and developed relatively slowly from 60 to 90 d. Elevating the w/b ratio from 0.4 to 0.45 and 0.5 increased the drying shrinkage at 90 d by approximately 9% and 30%, respectively. Water evaporation was the essential cause of the concrete’s drying shrinkage [58]. Because of the large pore volume and the great number of pores, additional moisture loss occurred at high w/b ratios [59,60].

In addition, the increased Na_2_O/b ratio led to great drying shrinkage. This result was consistent with previous studies [61]. As shown in Figure 8, compared with that of MCGC0.45-6-50, the drying shrinkage of the specimens with 8% and 10% Na_2_O/b ratios at 90 d increased by 15% and 44%, respectively. This result arose due to the accelerated hydration reaction, resulting in an increased number of gel pores and mesopores. References [62,63] noted that mesopores and gelled pores were the major causes of great drying shrinkage.

Additionally, a high Na_2_O/b ratio could lead to very early gel precipitation, which hindered the later hydration reaction and enlarged the porosity [64]. In short, increasing the Na_2_O/b ratio adversely affected the drying shrinkage. In summary, the drying shrinkage rate exhibited an increasing trend with increased Na_2_O/b ratios.

In contrast, a high MK/b ratio was of special interest for improving drying shrinkage. Figure 8 shows that as the replacement rate of slag increased from 30% to 50% and 70%, the drying shrinkage rates at 90 d decreased to 801 × 10^−6^ and 667 × 10^−6^, respectively. This result arose because the C-A-S-H gel formed in the hydration reaction filled the pores of the N-A-S-H gel to enhance the concrete’s compactness [65,66]. Moreover, the elevated MK/b ratio increased the Al/Si ratio, which could enhance the volume stability and reduce the sensitivity to water loss which had a good effect on reducing drying shrinkage [67,68].

### 3.3. Analysis and Establishment of the Drying Shrinkage Model for the MCGC

To date, many existing drying shrinkage prediction models are aimed at conventional concrete, such as ACI-209 [69], CEB-FIP [70], GL-2000 [71], and the Model of the China Academy of Building Research (CABR) [72]. Table 5 presents a summary of the prediction models for conventional concrete’s drying shrinkage. Furthermore, their prediction results were affected by a variety of parameters like the cement type, curing conditions, sand ratio, the concrete’s compressive strength, and specimen size, but their applicability was recognized.

#### 3.3.1. Applicability of the Existing Prediction Models for MCGC

**Figure 9:**
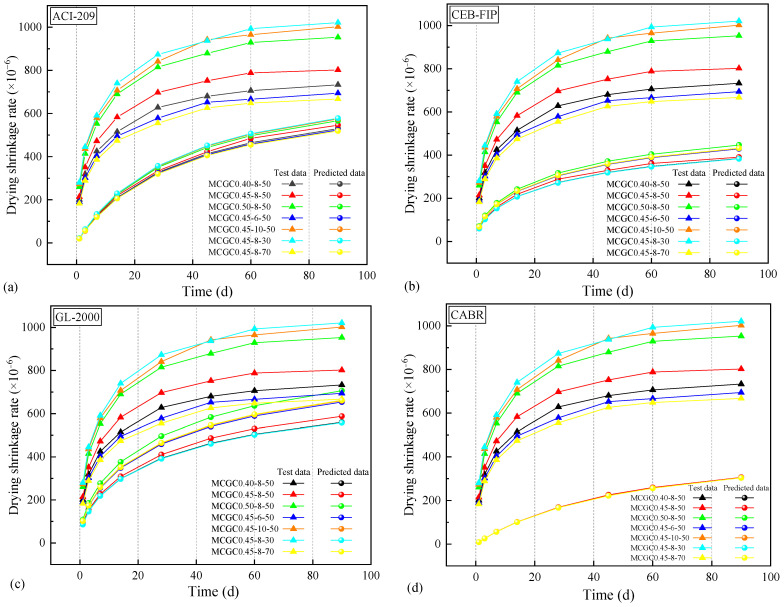
Comparison between the experimental and predicted drying shrinkage rates of the MCGCs: (**a**) ACI-209; (**b**) CEB-FIP; (**c**) GL-2000; (**d**) CABR.

Figure 9 displays a comparison between the predicted MCGC’s drying shrinkage rate based on the existing model and the measured value. The predicted data of the common models were significantly lower than the experimental results of MCGC. The predicted values of the GL-2000 model were the closest to the experimental data, followed by those of the ACI-209 model. However, the GL-2000 model clearly defined the relationship between the drying shrinkage and the strength, which was not applicable to the MCGC developed here. For instance, MCGC0.45-8-70 exhibited lower strength and drying shrinkage behavior than MCGC0.45-8-30. Moreover, the drying shrinkage errors of the CEB-FIP model and CABR model at 90 d were 235 × 10^−6^~638 × 10^−6^ and 363 × 10^−6^~717 × 10^−6^, respectively, which seriously underestimated MCGC’s drying shrinkage. As Table 5 displays, the conventional concrete prediction models for drying shrinkage considered factors such as specimen size, cement type, compressive strength, and curing conditions. Nevertheless, for MCGC the existing models did not fully consider all the factors in this study, which reduced the prediction accuracy.

#### 3.3.2. Establishment of the Prediction Model of Drying Shrinkage on the Basis of the MCGC

According to Section 3.3.1, the ACI-209 model has the closest prediction data to the test results and is widely used. However, considering the multiple control factors in the MCGC developed in this study, the model has to be further improved. By observing the function of the ACI-209 model in Table 5, the simplification was given by the following equation:

(1) $$\varepsilon_{sh}\left(t\right)=A\times {\left(\frac{t}{t+B}\right)}^{C}$$

As shown in Table 6, the value of parameter *C* was approximately 0.4. Furthermore, parameter *B* had little effect on the drying shrinkage value and varied irregularly. Therefore, the ACI-209 model could be substituted for 35. Table 6 shows that parameter A changed greatly, mainly due to the different values of the w/b, Na_2_O/b, and MK/b ratios. This finding indicated that the determination of A had to introduce the factors involved in this study. The analysis revealed a prominent numerical correlation between the parameters. The influence coefficient was defined as $\gamma_{\eta }$, which could be normalized as follows:

(2) $$\gamma_{\omega }=aw+b$$

(3) $$\gamma_{\theta }=cq+d$$

(4) $$\gamma_{m}=em+f$$

(5) $$\gamma_{\eta }=\gamma_{\omega }\gamma_{\theta }\gamma_{m}$$

where $\gamma_{\omega }$ is the influence coefficient of the w/b ratio, w is the w/b ratio, $\gamma_{\theta }$ is the influence coefficient of the Na_2_O/b ratio, *θ* is the Na_2_O/b ratio, $\gamma_{m}$ is the influence coefficient of the MK/b ratio, *m* is the MK/b ratio, *a*, *b*, *c*, *d*, *e*, and *f* are constants, and $\gamma_{\eta }$ is the influence coefficient of the MCGC. By introducing $\gamma_{\eta }$, $\varepsilon_{sh}(t)$ could be expressed as follows:

(6) $$\varepsilon_{sh}(t)=780\times \gamma_{cp}\gamma_{\lambda }\gamma_{h}\gamma_{s}\gamma_{\varphi }\gamma_{c}\gamma_{a}\gamma_{\eta }\times {\left(\frac{t}{t+35}\right)}^{0.4}\times 10^{-6}$$

According to the test data, the influence coefficients of specimens at different ages were calculated by Equation (6) and then linear regression analyses were performed to obtain the $\gamma_{\eta }$ value for each specimen, as displayed in Table 6. To enable $\gamma_{\eta }$ to generally reflect various factors it was necessary to carry out nonlinear regression analysis with the w/b, Na_2_O/b, and MK/b ratio values to calculate constant values. Therefore, the ACI-209 model was modified according to the experimental results and a prediction model was proposed for MCGC’s drying shrinkage behavior:

(7) $$\varepsilon_{sh}(t)=\varepsilon_{shu}\beta \left(t\right)=780\times \gamma_{cp}\gamma_{\lambda }\gamma_{h}\gamma_{s}\gamma_{\varphi }\gamma_{c}\gamma_{a}\gamma_{\eta }\times {\left(\frac{t}{t+35}\right)}^{0.4}\times 10^{-6}$$

(8) $$\gamma_{\eta }=(0.13w+0.0175)\times (0.083q+0.94)\times (-0.07m+14.53)$$

#### 3.3.3. Verification of a Prediction Model of Drying Shrinkage for MCGC

**Figure 10:**
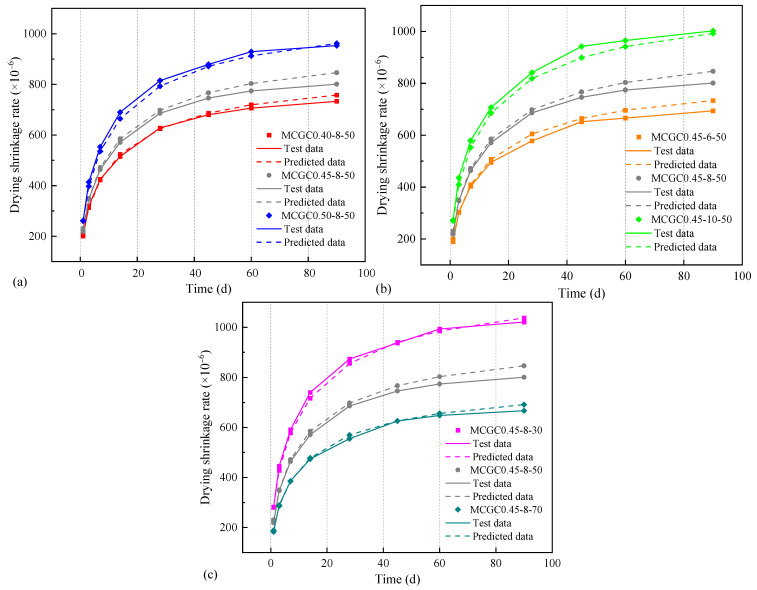
Model validation of the prediction model developed for the MCGCs: (**a**) specimens with different w/b ratios; (**b**) specimens with different Na_2_O/b ratios; (**c**) specimens with different MK/b ratios.

Figure 10 displays a comparison between the prediction model and the experimental data of MCGC’s drying shrinkage. As shown in Figure 10a–c, the drying shrinkage prediction model based on MCGC which was developed in this study has a better prediction effect than the existing prediction model. R^2^ was close to 0.94, which could effectively reflect the influence of the three control factors on drying shrinkage. Moreover, the model showed the development of MCGC’s drying shrinkage. The growth rate of drying shrinkage was relatively fast before 60 d and then gradually slowed, which was in agreement with the drying shrinkage development characteristics of geopolymer concrete. Overall, the model developed in this study could accurately fit the drying shrinkage curve of the experiment and had preferable practicability and reliability. Despite the high predictive accuracy of the model, further enhancements were necessary due to the diversity of pretreatment methods for ceramsite and the limitations of factors considered by the MCGCs.

## 4. Conclusions

This study compared the effects of various pretreatment methods on ceramsite and further investigated the influences of the w/b, Na_2_O/b, and MK/b ratios on MCGCs’ drying shrinkage and compressive strength. The following conclusions are according to the results and discussion of the test:

(1) Both the prewetting and silicone resin modified surfaces of the ceramsite increased the slump of the concrete. Prewetting aggravated the drying shrinkage and weakened the compressive strength before 7 d. In contrast, the silicone resin modified surface improved the compressive strength to a certain extent. Although the two samples exhibited similar drying shrinkage rates at 90 d, MCGC0.45-8-50 reduced by 16% and 10% at 3 and 7 d, respectively, indicating that the silicone resin modified surface favored the volume stability of the concrete.
(2) Increases in the w/b and MK/b ratios decreased the compressive strength. However, 8% Na_2_O/b enhanced the compressive strength, while a relatively low compressive strength was acquired with 10% Na_2_O/b.
(3) A high w/b/ratio enhanced the drying shrinkage characteristics. Concurrently, an analogous trend was observed with an increasing Na_2_O/b ratio. MCGC’s drying shrinkage rate peaked at 0.45-10-50 at 90 d among all the groups. In contrast, a low drying shrinkage behavior was observed when the MK/b ratio increased from 30% to 70%. Therefore, among all the factors, only a high MK/b ratio could significantly improve the drying shrinkage.
(4) The existing prediction models for drying shrinkage were not fully applicable to MCGC. Among the common drying shrinkage prediction models, the order of prediction accuracy was GL-2000 > ACI-209 > CEB-FIP > CABR. Although GL-2000 had a high prediction accuracy, it clearly stipulated the relationship between strength and shrinkage. This approach was not applicable to the MCGC proposed in this study. Therefore, the ACI-209 model was modified by considering the w/b, Na_2_O/b, and MK/b ratios to establish a drying shrinkage prediction model suitable for MCGC. The prediction results of the modified model were increasingly accurate. However, due to the diversity of pretreatment methods for ceramsite and the limitations of the considered factors, the model had potential for additional enhancements.

## Figures and Tables

**Table 1:** Mix proportions of the specimens.

Group	No.	Pretreatment	Shale Ceramsite, kg/m^3^	Sand, kg/m^3^	Binder, kg/m^3^		w/b	Na_2_O/b
					Slag	MK		
Group 1	UCGC0.45-8-50	U	461	750	240	240	0.45	8
	WCGC0.45-8-50	W	461	750	240	240	0.45	8
	MCGC0.45-8-50	M	461	750	240	240	0.45	8
Group 2	MCGC0.40-8-50	M	461	750	240	240	0.40	8
	MCGC0.45-8-50	M	461	750	240	240	0.45	8
	MCGC0.50-8-50	M	461	750	240	240	0.50	8
	MCGC0.45-6-50	M	461	750	240	240	0.45	6
	MCGC0.45-10-50	M	461	750	240	240	0.45	10
	MCGC0.45-8-30	M	461	750	144	336	0.45	8
	MCGC0.45-8-70	M	461	750	336	144	0.45	8

Notes: UCGC, WCGC, and MCGC represent untreated ceramsite geopolymer concrete, prewetted ceramsite geopolymer concrete, and modified ceramsite geopolymer concrete, respectively. U, W, and M represent the untreated, prewetted, and silicone resin modified surfaces, respectively.

**Table 2:** Physical properties of the shale ceramsite.

Grain Size, mm	Bulk Density, kg/m^3^	Cylinder Compressive Strength, MPa	Appearance Density, kg/m^3^	Absorption Rate, %
5–16	548	4.6	1347	8.6

**Table 3:** Technical indexes of silicone resin.

pH	Solid Content, %	Density, g/cm^3^	Water Absorption in 48 h, %	Penetration Depth, cm
10.50	35.00	1.07	12.00	3.00

**Table 4:** Main chemical compositions of MK and slag (wt.%).

Materials	SiO_2_	Al_2_O_3_	CaO	MgO	SO_3_	Fe_2_O_3_	TiO_2_	Na_2_O	K_2_O	L.O.I.
Slag	33.21	15.76	37.05	8.51	2.57	0.41	1.35	0.29	0.32	0.46
MK	49.67	42.54	0.19	0.14	0.27	0.68	0.63	0.12	0.18	0.62

Notes: L.O.I. stands for loss on ignition.

**Table 5:** Prediction models for drying shrinkage.

Model	Equation	Time-Varying Function	Correlation Parameters
ACI-209R-92	$\varepsilon_{sh}\left(t\right)=\frac{t}{f+t}\varepsilon_{shu}$ $\varepsilon_{shu}=\gamma_{cp}\gamma_{\lambda }\gamma_{h}\gamma_{s}\gamma_{\varphi }\gamma_{c}\gamma_{a}\times 780\times 10^{-6}$	$\frac{t}{35+t},\frac{t}{55+t}$	*t*
CEB-FIP	$\varepsilon_{cs}(t)=\varepsilon_{cso}\beta_{s}(t)=\varepsilon_{s}(f_{c28})\beta_{RH}\beta_{s}(t)$ $\varepsilon_{s}(f_{c28})=(160+10\beta_{sc}(9-0.1f_{c28}))\times 10^{-6}$ $\beta_{RH}=\left\{\begin{array}{cc} 1.55\left(1-{\left(\frac{RH}{100}\right)}^{3}\right) & 40\%\leq RH\leq 99\%\\ -0.25 & RH>99\% \end{array}\right.$ $\beta_{s}\left(t\right)=\sqrt{\frac{t}{0.035h^{2}+t}}$	$\beta_{s}(t,t_{s})=\sqrt{\frac{t}{0.035h^{2}+t}}$	*t*, *h*
GL-2000	$\varepsilon_{sh}\left(t\right)=\varepsilon_{shu}k_{h}\beta \left(t\right)$ $\varepsilon_{shu}=1000K{\left(\frac{30}{f_{cm}}\right)}^{0.5}\times 10^{-6}$ $k_{h}=1-1.18h^{4}$ $\beta \left(t\right)=\sqrt{\frac{t-t_{c}}{t-t_{c}+0.15{\left(V/S\right)}^{2}}}$	$\beta \left(t\right)=\sqrt{\frac{t}{t+0.15{\left(V/S\right)}^{2}}}$	*t*, *V*, *S*
CABR	$\varepsilon \left(t\right)=\varepsilon {\left(t\right)}_{0}\cdot \beta_{1}\cdot \beta_{2}\cdot \beta_{3}\cdot \beta_{4}\cdot \beta_{5}$ $\begin{array}{cc} \varepsilon {\left(t\right)}_{0}=\frac{t}{152.79+3.27t}\times 10^{-3} & \left(ordinary\;concrete\right) \end{array}$ $\begin{array}{cc} \varepsilon {\left(t\right)}_{0}=\frac{t}{120.23+2.26t}\times 10^{-3} & \left(lightweight\;concrete\right) \end{array}$	$\varepsilon {\left(t\right)}_{0}=\frac{t}{120.23+2.26t}\times 10^{-3}$	*t*

Notes: *RH* represents relative humidity.

**Table 6:** Values of the fitting parameters and influence coefficients of the MCGCs.

	$\varepsilon_{sh}(t)\;=\;A\times {\left(t/t\;+\;B\right)}^{C}$			Influence Coefficient of the MCGC	
No.	*A* (×10^−6^)	*C*	R^2^	$\gamma_{\eta }$	R^2^
MCGC0.40-8-50	840	0.3927	0.98682	1.15915	0.98642
MCGC0.45-8-50	920	0.39469	0.98611	1.24338	0.97837
MCGC0.50-8-50	1094	0.39382	0.98508	1.41553	0.98279
MCGC0.45-6-50	795	0.39115	0.98666	1.10996	0.99163
MCGC0.45-10-50	1137	0.40677	0.97485	1.46005	0.98563
MCGC0.45-8-30	1169	0.39181	0.98529	1.48491	0.98570
MCGC0.45-8-70	759	0.40446	0.97836	1.07752	0.97468

## Data Availability

The original contributions presented in this study are included in the article. Further inquiries can be directed to the corresponding author.

## Abbreviations

The following abbreviations are used in this manuscript:

MCGC	modified ceramsite geopolymer concrete
MK	metakaolin
L.O.I	loss on ignition
w/b	water/binder ratio
Na_2_O/b	sodium oxide concentration ratio
MK/b	metakaolin content ratio
*RH*	relative humidity
